# Supplementary material for: PD-L1 deletion or blockade regulate macrophage antigen presentation and checkpoint molecule surface levels
Source: bioRxiv. 2026 Jun 29:2026.06.23.734016. Preprint. [Version 1] doi: 10.64898/2026.06.23.734016 (PMC13345199; doi:10.64898/2026.06.23.734016)
Supplement: Supplement 1 [file NIHPP2026.06.23.734016v1-supplement-1.pdf]

bioRxiv preprint doi: https://doi.org/10.64898/2026.06.23.734016; this version posted June 29, 2026. The copyright holder for this preprint (which was not certified by peer review) is the author/funder, who has granted bioRxiv a license to display the preprint in perpetuity. It is made available under a CC-BY 4.0 International license.

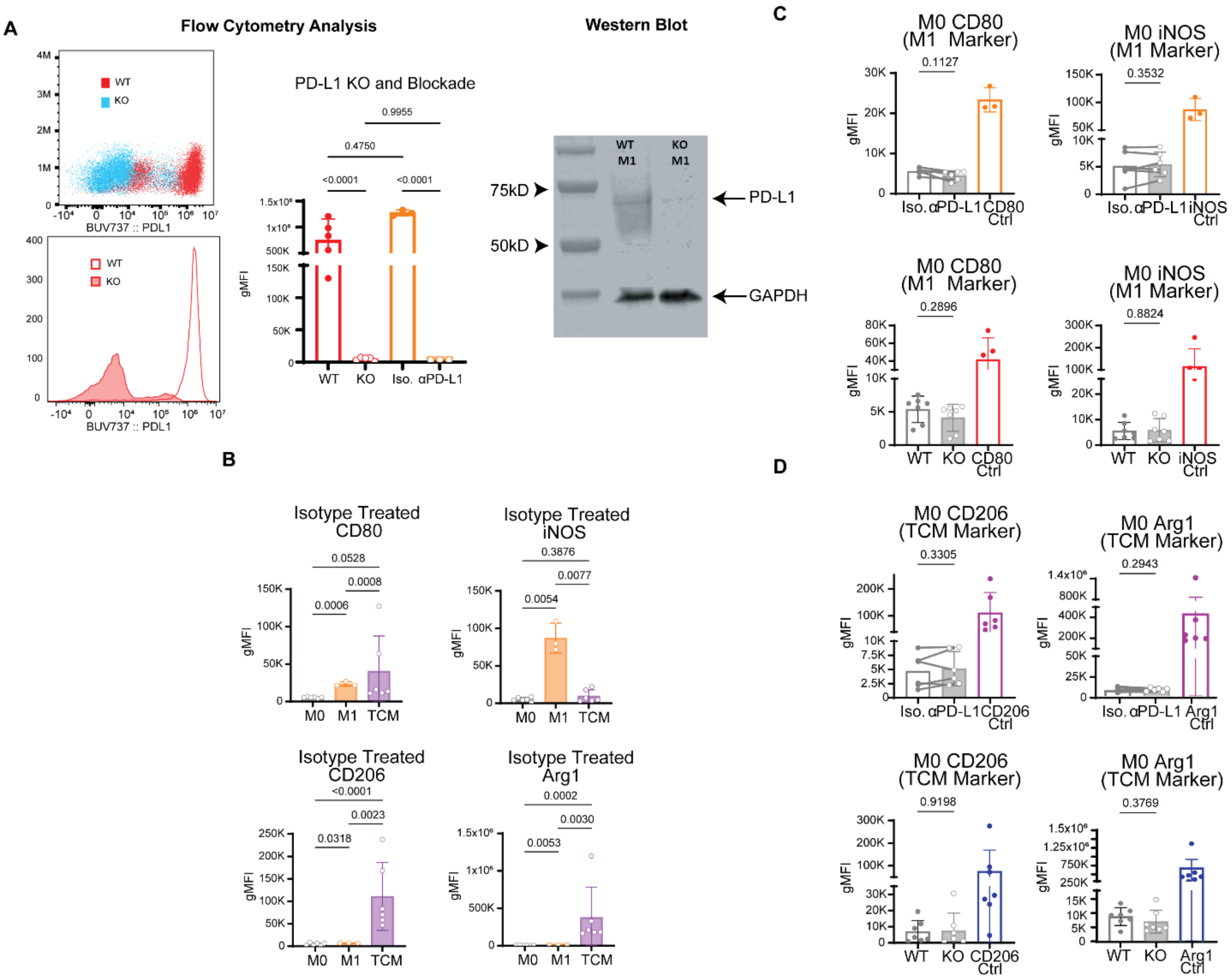

Supplementary Figure 1

(A) PD-L1 knockout and blockade confirmation through flow cytometry (left figure) and western blot confirmation of PD-L1 knockout. All M1 polarized. Demonstrating compete knockout and complete blockade of surface PD-L1. Statistical analysis was performed using a Lognormal ordinary one-way ANOVA with Tukey's multiple comparisons Test. (B) Baseline expression of polarization panel in Isotype treated BMDMs. CD80 TCM expression appears higher than M1 BMDMs due to experimental day variability resulting in outliers. Experimental day matched samples result in M1 BMDMs with the highest expression. Statistical analysis was performed using Mixed-effects analysis with Tukey's multiple comparisons. (C) M1 marker expression of naïve BMDMs WT/KO and Iso/aPD-L1. (D) TCM marker expression of naïve BMDMs WT/KO and Iso/aPD-L1. The remaining statistical analysis was performed with Welch's T test analysis of Log10 transformed data for WT/KO analysis and Ratio paired T test for Iso/aPD-L1 analysis.

bioRxiv preprint doi: https://doi.org/10.64898/2026.06.23.734016; this version posted June 29, 2026. The copyright holder for this preprint (which was not certified by peer review) is the author/funder, who has granted bioRxiv a license to display the preprint in perpetuity. It is made available under a CC-BY 4.0 International license.

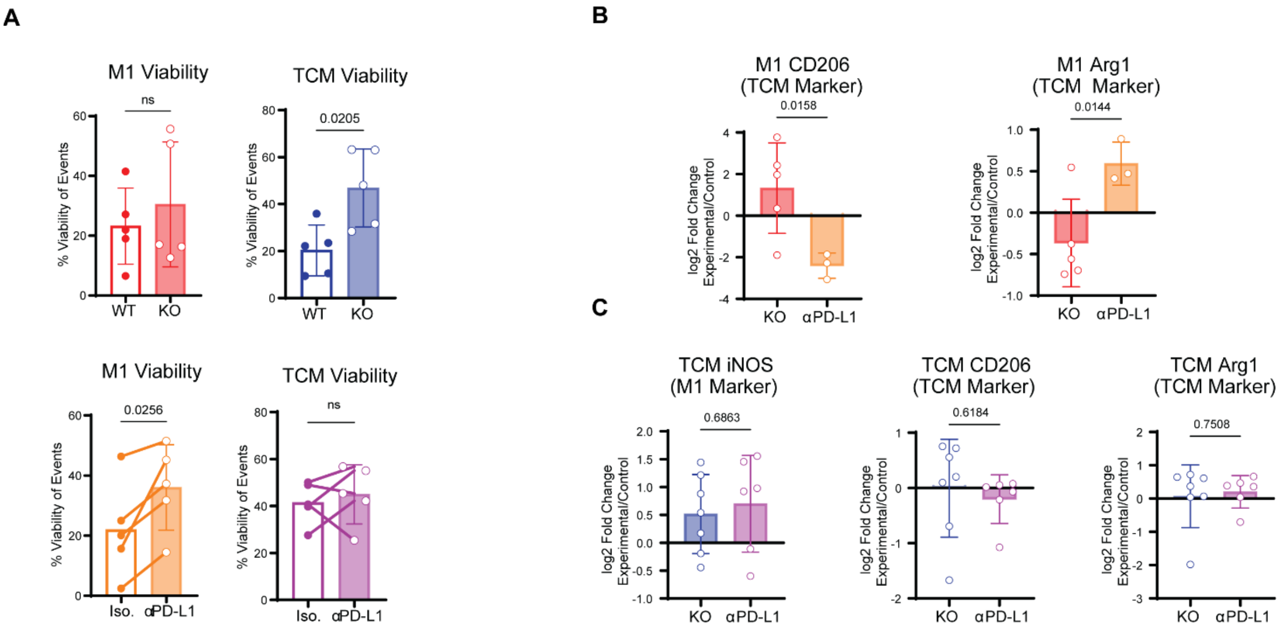

Supplementary Figure 2

(A) Cell viability analyzed using a viability dye as the fraction of viable cells of the total cell population lifted from the well. (N=5 mice from 1 experiment) Statistical analysis performed using Welch's T test for WT/KO and Paired T test for Iso/aPD-L1. (B) Log2 fold change of KO/WT and aPD-L1/iso of CD206 and Arg1 expression in M1 polarized BMDMs. (N=3-5 mice from 1-3 experiments) Statistical analysis performed using Welch's T test. (C) Log2 fold change of KO/WT and aPD-L1/iso of iNOS, CD206, and Arg1 expression in TCM polarized BMDMs. (N=6-7 mice from 3-5 experiments) Statistical analysis performed using Welch's T test.

bioRxiv preprint doi: https://doi.org/10.64898/2026.06.23.734016; this version posted June 29, 2026. The copyright holder for this preprint (which was not certified by peer review) is the author/funder, who has granted bioRxiv a license to display the preprint in perpetuity. It is made available under a CC-BY 4.0 International license.

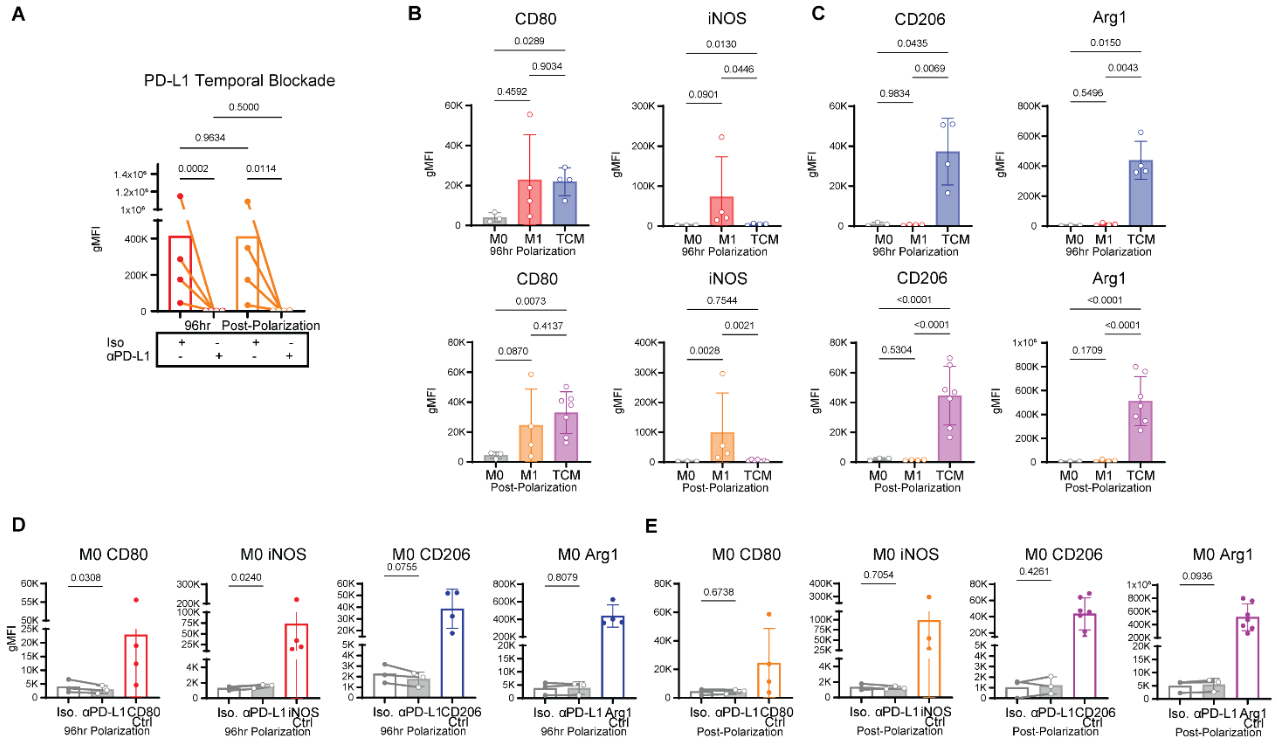

Supplementary Figure 3

(A) Flow cytometry analysis performed to confirm blockade of PD-L1 in the 96hr and post-polarization M1 polarized treatment groups. (N=4 mice from 2 experiments) Statistical analysis was performed using a Lognormal ordinary one-way ANOVA with Tukey's multiple comparisons Test. (B) Baseline expression of M1 polarization markers in 96hr and post-polarization groups. (N=3-7 mice from 1-4 experiments.) Statistical analysis was performed using Mixed-effects analysis with Tukey's multiple comparisons. (C) Baseline expression of TCM polarization markers in 96hr and post-polarization groups. (N=3-7 mice from 1-4 experiments.) Statistical analysis was performed using Mixed-effects analysis with Tukey's multiple comparisons. (D) Polarization marker expression of naïve BMDMs in the 96hr treatment group. (E) Polarization marker expression of naïve BMDMs in the post-polarization treatment group. The remaining statistical analysis was performed with Ratio paired T test.

bioRxiv preprint doi: https://doi.org/10.64898/2026.06.23.734016; this version posted June 29, 2026. The copyright holder for this preprint (which was not certified by peer review) is the author/funder, who has granted bioRxiv a license to display the preprint in perpetuity. It is made available under a CC-BY 4.0 International license.

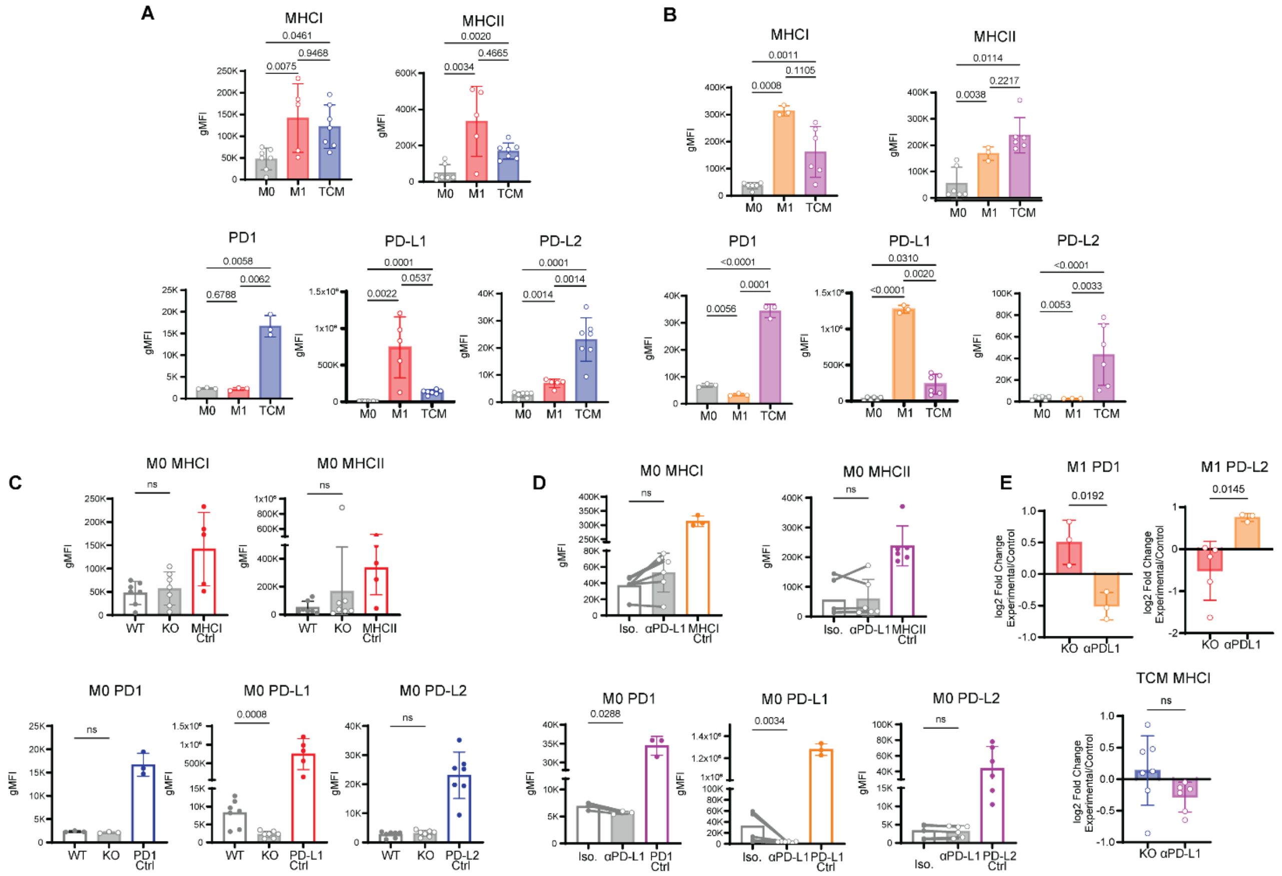

Supplementary Figure 4

(A) Baseline expression of antigen presentation and checkpoint markers of WT BMDMs. (N=3-7 from 1-4 experiments.) Statistical analysis was performed using Mixed-effects analysis with Tukey's multiple comparisons. (B) Baseline expression of antigen presentation and checkpoint markers of Isotype treated BMDMs. (N=3-6 from 1-4 experiments.) Statistical analysis was performed using Mixed-effects analysis with Tukey's multiple comparisons. (C) Antigen presentation and checkpoint marker expression of WT/KO naïve BMDMs. (N=3-7 from 1-4 experiments). (D) Antigen presentation and checkpoint marker expression of Iso/aPD-L1 naïve BMDMs. (N=3-6 from 1-4 experiments). C and D statistical analysis performed with Welch's T test analysis of Log10 transformed data for WT/KO analysis and Ratio paired T test for Iso/aPD-L1 analysis. (E) Log2 fold change of KO/WT and aPD-L1/iso of PD1 and PD-L2 expression in M1 polarized BMDMs. (N=3-5 mice from 1-3 experiments) and MHCI expression in TCM polarized BMDMs. (N=5-7 mice from 3-4 experiments) Statistical analysis performed using Welch's T test.
